# Optical clearing of apple tissues for in vivo imaging of the pathogenic behavior of the fungus *Botryosphaeria dothidea* on host surfaces

**DOI:** 10.1186/s42649-025-00110-w

**Published:** 2025-05-01

**Authors:** Ki Woo Kim, Eun Woo Park

**Affiliations:** 1 Department of Agricultural Biotechnology, Seoul National University, Seoul, 08826 Republic of Korea; 2 Department of Forest Ecology and Protection, Kyungpook National University, Sangju, 37224 Republic of Korea; 3 Tree Diagnostic Center, Kyungpook National University, Sangju, 37224 Republic of Korea; 4 Field Support Education Division, EPINET, Pyeongchang, 25354 Republic of Korea

**Keywords:** Anthocyanin, Appressoria, Germination, Pigment, Scattering

## Abstract

Optical clearing of apple tissues was performed to observe the pre-penetration behavior of *Botryosphaeria dothidea*. Mature red fruits and two-year-old twigs were artificially inoculated with the fungal conidia. Fruit epidermis and twig cork tissues were excised and immersed overnight in an ethanol-chloroform solution amended with trichloroacetic acid. Lactophenol cotton blue was used to stain the fungus on the host surfaces. The morphology and behavior of the inoculated *B. dothidea* could be clearly observed in the two types of optically cleared specimens. The conidia showed either monopolar or bipolar germination, leading to the emergence of germ tubes from one or both conidial ends. Conidia formed appressoria at the terminal ends of germ tubes. They appeared round, hook-shaped, and irregular-shaped in two-dimensional light micrographs. Multiple appressoria were observed on the suberized phellem cells in twig lenticels. These results suggest that the optical clearing technique and fungal staining were effective in partially decolorizing apple tissues and revealing the fungal structures on the host surfaces.

## Introduction

Plants possess innate characteristics manifested by cell walls, fibers, and pigments. These entities deteriorate light microscopy (LM) resolution by absorbing and scattering visible light, leading to a refractive index mismatch between cellular components (Hériché et al. 2022; Yu et al. 2018). Observing plant specimens using conventional LM without sectioning or pigment removal is challenging. Optical clearing techniques represent a series of chemical steps that render a bulk specimen transparent (Ariel 2017; Kim 2024). Various optical clearing techniques have been primarily developed for animal tissues to achieve uniformity of refractive indices throughout the specimen (Yu et al. 2018).

Several clearing techniques have been implemented to observe fungal hyphae in plant tissues. Bread wheat (*Triticum aestivum*) leaves infected with *Puccinia* or *Fusarium* were cleared by immersing in an ethanol-chloroform solution containing 0.15% trichloroacetic acid (Knight and Sutherland 2011). Maize (*Zea mays*) leaves were transferred to a Sca*l*eP solution containing urea, glycerol, and Triton X-100 in sterile water (Warner et al. 2014). These optically cleared specimens were observed using fluorescence microscopy, enabling deeper imaging of hyphal growth in plant tissues.

The ascomycete fungus *Botryosphaeia dothidea* causes canker and dieback diseases in various trees belonging to over 24 plant genera worldwide, including *Malus* (Lee et al. 2023; Marsberg et al. 2017). It is also the causal pathogen of apple white rot, manifested by light brown, concentric ring rots on the slightly sunken fruits and blisters on stems (Kim et al. 2005; Sutton 1991). The fungal conidia germinate, form appressoria on apple fruits, and enter microcracks, as revealed by scanning electron microscopy (Kim et al. 1999). An infection model has been developed to control the disease with fewer fungicide applications compared to calendar-based conventional control regimes by observing and modeling the impacts of temperature and wetness periods on the conidial germination and appressorial formation on apple fruits (Kim et al. 2005). Although fungal infections manifest prominently on host plants under stress, the significance of latent fungal infections is considerable, as they suggest that the pathogen may evade detection by quarantine systems (Marsberg et al. 2017). The glyoxysomal nature of microbodies complexed with lipid globules was suggested as the cellular basis of the latent infection of *B. dothidea* (Kim et al. 2001; 2004).

However, little information is available regarding observations of the pathogenic behavior of *B. dothidea* on host surfaces using conventional upright LM. Optical clearing techniques are essential for the removal of pigments and the modification of cellular components. The objectives of this study were to (i) evaluate a clearing solvent mixture suitable for apple tissues and (ii) observe the fungal conidial germination and appressorial formation on the host surfaces.

## Materials and methods

### Fungal inoculum preparation

**Fig. 1:**
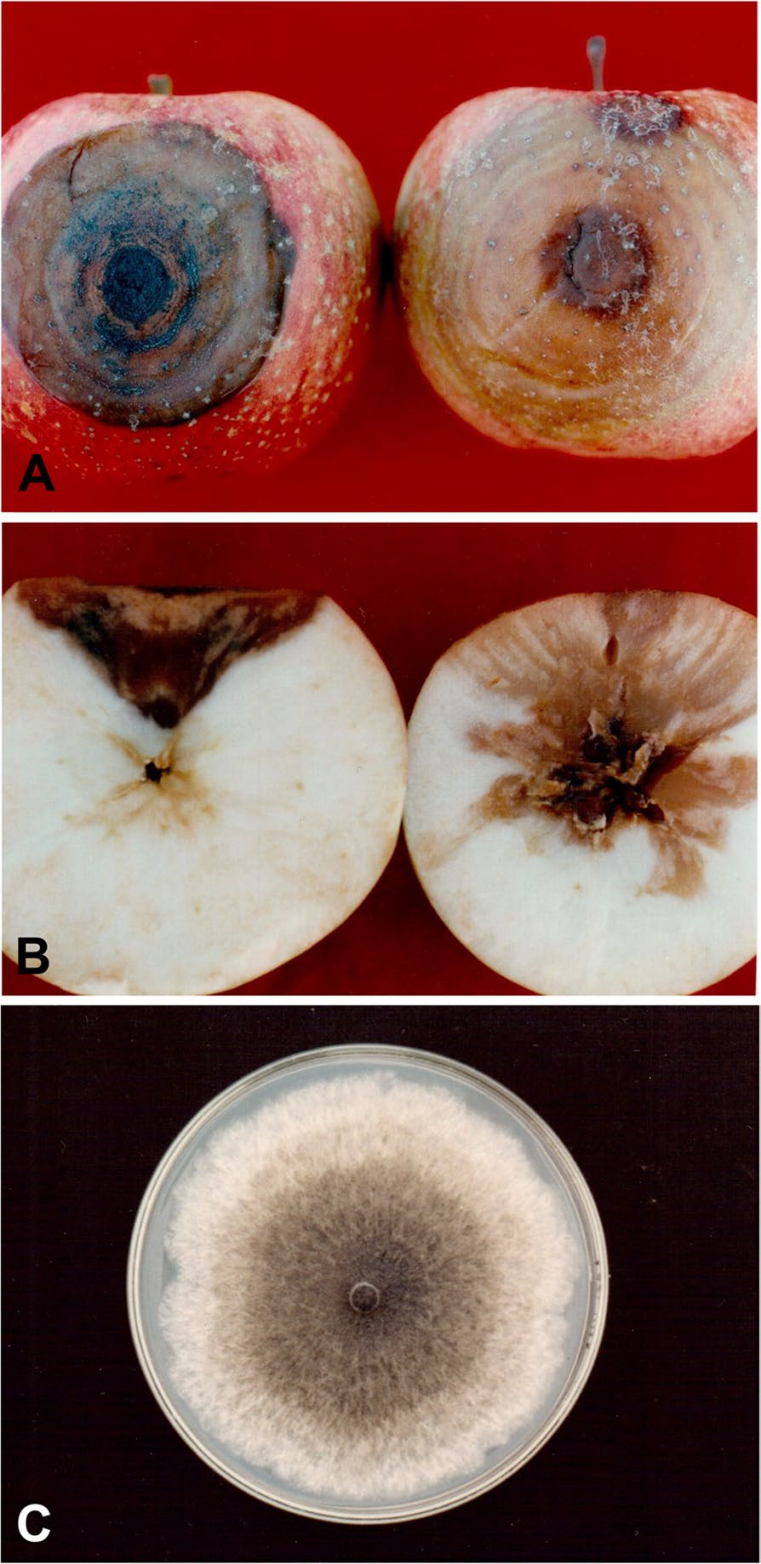
Photographs of naturally infected apple fruits and *Botryosphaeria dothidea*. (**A**) Bitter rot caused by *Colletotrichum* (left) featuring dark brown and sunken lesions; white rot caused by *Botryosphaeria* (right) featuring light brown and slightly sunken lesions. (**B**) Internal symptoms of bitter rot (left) showing V-shaped lesions and white rot (right) showing lesion extension to the core. (**C**) *B. dothidea* culture on potato dextrose agar medium

Naturally infected apple fruits were collected from an apple orchard in Suwon (37°17′28"N and 127°00′32"E), Korea (Fig. 1A). An isolate of *B. dothidea* (BD-3) was obtained from an apple fruit exhibiting typical white-rot symptoms such as a light brown, concentric ring rot on slightly sunken fruit tissues (Kim et al. 1999). The lesions extended to the core in the fruits (Fig. 1B). The fungal isolate was grown on acidic (pH 4 to 5) potato dextrose agar (Difco Laboratories, Detroit, MI, USA) amended with 100 mg/ml streptomycin sulfate (Chong Kun Dang Pharmaceutical Co., Seoul, Korea) to prevent bacterial contamination (Kim et al. 2016) (Fig. 1C). Barley grain media were inoculated with mycelial plugs of *B. dothidea* and incubated at 25 °C under continuous fluorescent light (Kim and Park 1998). Pycnidia were formed on the barley grains 3 days after inoculation. Conidia were harvested by immersing the inoculated barley grains in sterilized distilled water. The conidial suspension was filtered through two layers of cheesecloth to remove the mycelial fragments. The resulting suspension was adjusted to 1.0 × 10^5^ conidia/ml using a hemacytometer.

### Inoculation of apple tissues

Mature red fruits and two-year-old twigs were collected from ‘Fuji’ apple trees in the apple orchard. The specimens were washed with mild detergent to remove dirt and pesticide residues. They were then surface sterilized with 0.1% sodium hypochlorite solution for 2 min, rinsed with tap water, and left to dry in ambient air for 30 min (Kim et al. 2005). Inoculation sites on the fruit surface and twig lenticels were marked on each specimen for point inoculation. A conidial suspension droplet (approximately 200 μl) was placed on each inoculation site on the specimens using an adjustable micropipette (Kim et al. 2016). The fruits and twigs were immediately placed in a dew chamber at 25 °C under continuous dark conditions. Following a wetness period of 9 h, the specimens were removed from the dew chamber and processed for optical clearing to observe fungal behavior on the host.

### Optical clearing and fungal staining

Epidermis and cork tissues (each 1 × 1 cm with underlying tissue thickness of approximately 1 mm) were excised using a sterile razor blade from the inoculated fruits and twigs. The specimens were immersed overnight in an ethanol-chloroform (3:1; v/v) solution containing trichloroacetic acid at 0.15% (w/v) to decolorize specimens (Wolf and Fric 1981). They were then immersed in 10 ml of distilled water amended with two drops (approximately 50 μl each) of 0.1% lactophenol cotton blue for 10 min and observed using conventional upright LM (BH; Olympus Optical, Tokyo, Japan).

## Results

**Fig. 2:**
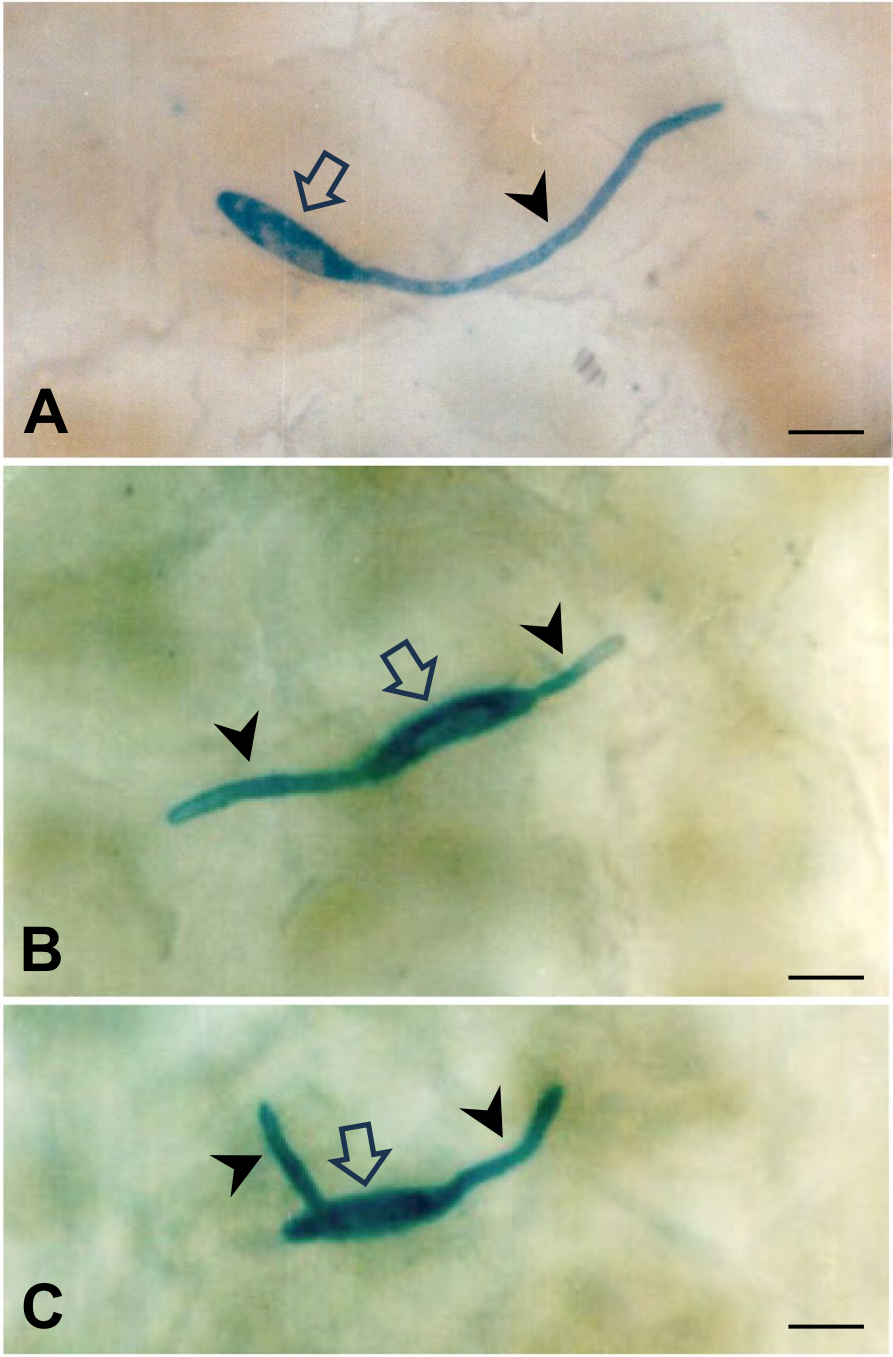
Light micrographs of conidial germination of *Botryosphaeria dothidea* on apple fruits. Epidermal peels were depigmented with organic solvents. Fungal structures were stained with lactophenol-cotton blue. **A** Polar germination. **B** Bipolar germination. **C** Bipolar orthogonal germination. An open arrow and a closed arrowhead indicate a conidium and a germ tube, respectively. Scale bars = 10 μm

The morphology of the inoculated fungus and its behavior could be clearly observed on the optically cleared apple fruit peels (Fig. 2). Fungal structures were specifically stained blue on the fruit surface. Conidia were spindle-shaped, tapering at both ends and approximately 20 μm in length. They exhibited either monopolar or bipolar germination, forming the germ tube from one or both conidial ends (Fig. 2A and B). Instances were also noted in which a germ tube emerged from a conidial polar end perpendicular to another germ tube emerging from the opposite end of the conidium (Fig. 2C).

**Fig. 3:**
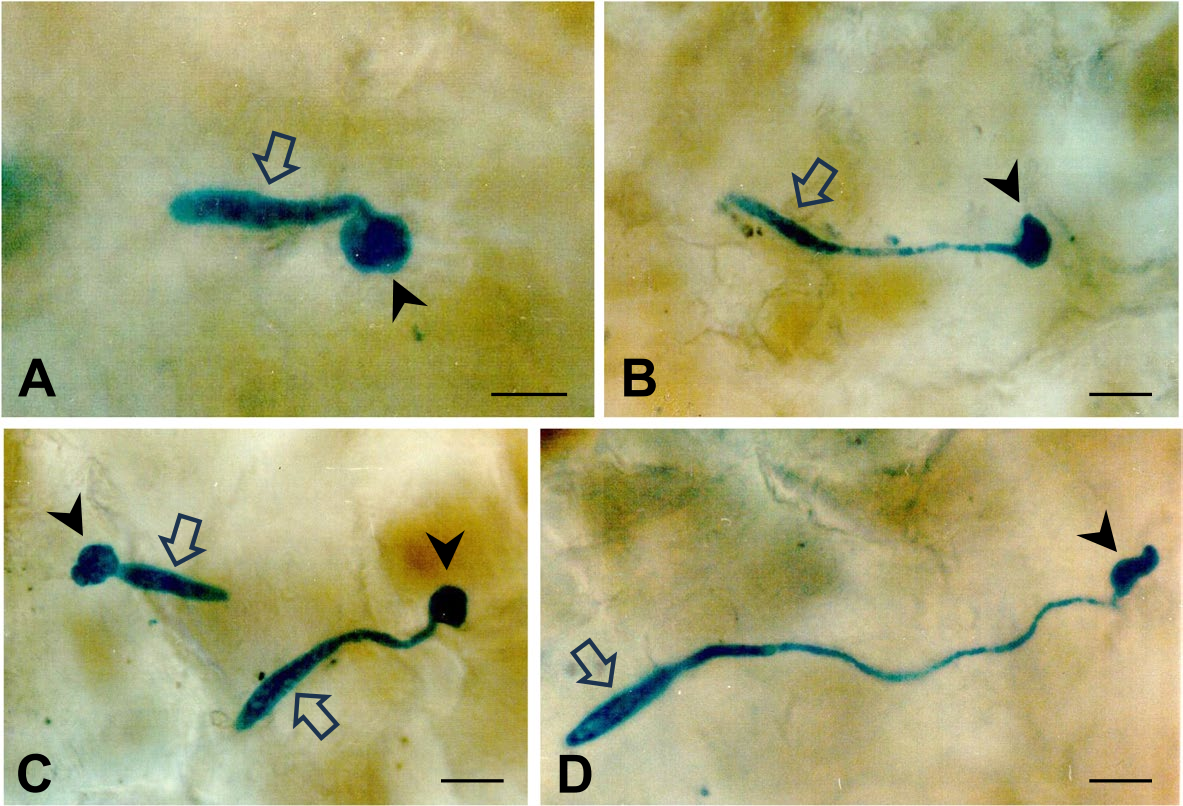
Light micrographs of conidial germination and appressorial formation of *Botryosphaeria dothidea* on apple fruits. Epidermal peels were depigmented with organic solvents. Fungal structures were stained with lactophenol-cotton blue. **A**–**D** Polar germination and appressorial formation with various germ tube lengths. An open arrow and a closed arrowhead indicate a conidium and an appressorium, respectively. Scale bars = 10 μm

After germination and germ tube elongation, conidia formed appressoria at the terminal ends of germ tubes (Fig. 3). Most appressoria were round (Fig. 3A and C); however, irregular-shaped appressoria were also found at the terminal germ tube ends (Fig. 3B and D).

**Fig. 4:**
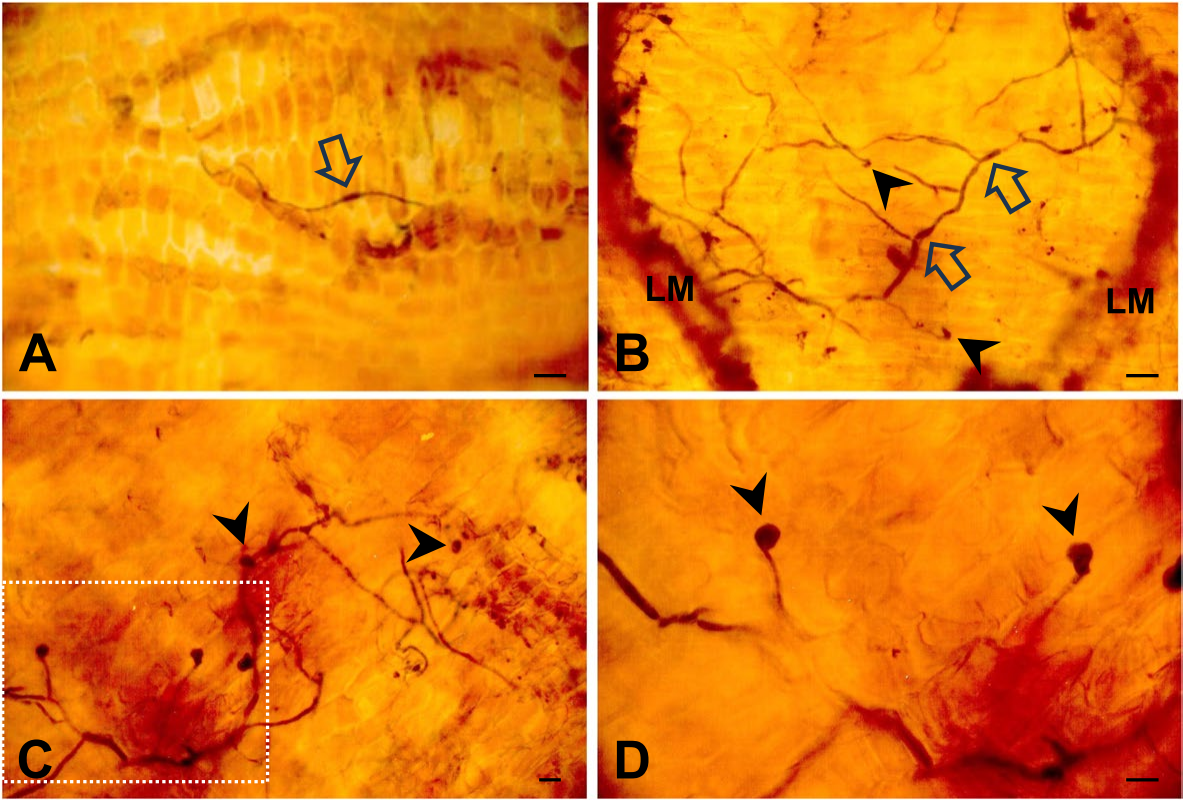
Light micrographs of conidial germination and appressorial formation of *Botryosphaeria dothidea* on apple twigs. Twig peels were depigmented with organic solvents. Fungal structures were stained with lactophenol-cotton blue. **A** Bipolar germination on the twig lenticel. **B** Bipolar germination and appressorial formation on the twig lenticel. LM = lenticel margin. Scale bars = 20 μm. **C** Bipolar germination and formation of multiple appressoria on the twig lenticel. **D** Magnified view of the dotted rectangle of (**C**). Note the round appressoria at the terminal ends of the germ tubes. An open arrow and a closed arrowhead indicate a conidium and an appressorium, respectively. Scale bars = 10 μm

Fungal structures could be observed on the optically cleared apple twig lenticels (Fig. 4). A fungal behavior similar to that on the fruit surface was observed on the twig lenticels. Nevertheless, the fungal structures were not as densely stained blue as those on the fruit surface. Bipolar germination of the fungal conidia occurred on the twig lenticels (Fig. 4A). The conidia formed appressoria at the terminal germ tube ends (Fig. 4B). Multiple appressoria were observed on the suberized phellem cells in the twig lenticels (Fig. 4C). A magnified view clearly showed round appressoria at the terminal ends of germ tubes (Fig. 4D).

## Discussion

This study demonstrated the pre-infection behavior of *B. dothidea* on apple organs, such as fruits and twigs, using conventional upright LM. Specimen immersion in the ethanol-chloroform solution containing trichloroacetic acid prior to LM observations effectively decolorized the tissues and could clearly reveal the fungal structures on the host surfaces. The coloration of apple skin results from chlorophyll and carotenoids in plastids and the phenolic pigments (anthocyanins, flavonols, and proanthocyanidins) in the vacuoles (Lancaster et al. 1994). Anthocyanins, red-colored pigments in the fruit epidermis, could be partially removed through extraction in a methanol-hydrogen chloride solution at 4 °C overnight (Bae et al. 2006). A protocol utilizing an ethanol-chloroform solution containing trichloroacetic acid was employed to observe the barley leaves infected with a powdery mildew fungus (Wolf and Frič 1981). According to the contemporary classification of optical clearing techniques for botanical applications, this protocol may be categorized under hydrophobic-related techniques due to amphiphilic and hydrophobic properties of ethanol and chloroform, respectively (Kim 2024).

In addition, lactophenol cotton blue, consisting of cotton blue (also referred to as methyl blue) and a solution of lactic acid, phenol, and glycerol, enabled the specific blue staining of fungal structures, including hyphae and appressoria on the host tissues. Cotton blue can stain chitin present in the fungal cell walls (Leck 1999). Given the biohazards of lactophenol in the stain solution, iodine glycerol was suggested as an alternative stain for the characterization of filamentous fungi (Shamly et al. 2014). Additional research is needed to assess integrated protocols for non-hazardous clearing and fungus-specific staining.

Different shapes of appressoria of *B. dothidea* were observed on the host surfaces. They appeared round, hook-shaped, and irregular-shaped in two-dimensional light micrographs. These morphological variations can arise from distinct developmental stages during appressorial formation or specific responses to different environmental stimuli, such as substrate composition and topography (Deising et al. 2000; Hoch et al. 1987). Various appressorial morphologies have been reported from saprophytic fungi and plant pathogenic fungi (Chethana et al. 2021). To our knowledge, this is the first report of the application of optical clearing to document the pre-penetration behavior of *B. dothidea* on apple fruits and twigs using light microscopy.

Lenticels are natural openings in the periderm distinguished from the phellem in having intercellular spaces and have been suggested to facilitate gas exchange (Angyalossy et al. 2016). The pores may serve as infection courts for various forest pathogens (Nemesio-Gorriz et al. 2019). Semi-thin sectioning revealed that the hyphae of artificially inoculated *B. dothidea* entered apple twigs through lenticels and grew both intercellularly and intracellularly (Han et al. 2016). Considering the conidial germination and the formation of multiple appressoria on apple twigs in this study, it was inferred that the fungus develops adhesion structures potentially for the initiation of penetration pegs to invade the lenticels. These results suggest that optical clearing techniques can be applied to elucidate the role of lenticels as potential infection courts in woody plants.

## Conclusion

A procedure of optical clearing and fungal staining was introduced to visualize pre-penetration behavior of *B. dothidea* on the surface of apple fruits and twigs. Ethanol-chloroform with trichloroacetic acid was effective for clearing tissues of apple fruits and twigs, and lactophenol cotton blue for staining fungal structures such as conidial germination, germ tube elongation, and appressorial formation on the surface of host tissues. Improvement of optical clearing techniques for woody tissues is necessary to further elucidate microbial growth in plants through in vivo imaging.

## Data Availability

Data and materials available on request.

## Abbreviations

LM: Light microscopy
RI: Refractive index
